# Alternative transient states and slow plant community responses after changed flooding regimes

**DOI:** 10.1111/gcb.14569

**Published:** 2019-01-30

**Authors:** Judith M. Sarneel, Mariet M. Hefting, George A. Kowalchuk, Christer Nilsson, Merit Van der Velden, Eric J. W. Visser, Laurentius A. C. J. Voesenek, Roland Jansson

**Affiliations:** ^1^ Landscape Ecology Group Department of Ecology and Environmental Sciences Umeå University Umeå Sweden; ^2^ Ecology & Biodiversity Institute of Environmental Biology Utrecht University Utrecht Netherlands; ^3^ Plant Ecophysiology Institute of Environmental Biology Utrecht University Utrecht Netherlands; ^4^ Department of Experimental Plant Ecology Institute for Water and Wetland Research Radboud University Nijmegen Netherlands

**Keywords:** alternative stable states, drought events, flood regime change, hydrological alterations, hysteresis, riparian vegetation, river restoration, species traits

## Abstract

Climate change will have large consequences for flooding frequencies in freshwater systems. In interaction with anthropogenic activities (flow regulation, channel restoration and catchment land‐use) this will both increase flooding and drought across the world. Like in many other ecosystems facing changed environmental conditions, it remains difficult to predict the rate and trajectory of vegetation responses to changed conditions. Given that critical ecosystem services (e.g. bank stabilization, carbon subsidies to aquatic communities or water purification) depend on riparian vegetation composition, it is important to understand how and how fast riparian vegetation responds to changing flooding regimes. We studied vegetation changes over 19 growing seasons in turfs that were transplanted in a full‐factorial design between three riparian elevations with different flooding frequencies. We found that (a) some transplanted communities may have developed into an alternative stable state and were still different from the target community, and (b) pathways of vegetation change were highly directional but alternative trajectories did occur, (c) changes were rather linear but faster when flooding frequencies increased than when they decreased, and (d) we observed fastest changes in turfs when proxies for mortality and colonization were highest. These results provide rare examples of alternative transient trajectories and stable states under field conditions, which is an important step towards understanding their drivers and their frequency in a changing world.

## Graphical abstract

To explore pathways of vegetation change after various flooding regime changes, we transplanted vegetation turfs full‐factorial between three riparian elevations (upland, middle and low elevation). Vegetation changes were directional and faster when flooding frequencies increased than when they decreased. After 19 growing seasons, we found indications for alternative stable states and alternative trajectories under field conditions, which is an important step towards understanding their drivers and their frequency in a changing world.

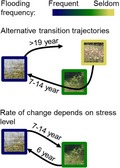

## 1 INTRODUCTION

Flood frequency worldwide is projected to increase in 42% and decrease in 18% of the global land area until the end of this century (Hirabayashi et al., 2013). At the same time, 90% of the global river volume is impacted by flow regulation (Grill et al., 2015). This has large consequences for riparian vegetation and associated ecosystem services like water purification, bank stability and biodiversity (Schulz et al., 2015). Natural flow regimes are therefore increasingly being restored, which often also induces large changes in the flow regime. In order to quantify and predict how and how fast changes in flooding affect plant community composition, more knowledge on the underlying processes is a priority. A difficulty in such studies (and similar ones in other systems) is determining the time needed for communities to adjust to new hydrological conditions (Walker et al., 2006), since this is often hampered by confounding factors (e.g. overruling effects of dispersal barriers) (Muller et al., 2014), insufficient time for follow‐up studies (Hasselquist et al., 2015) or limited spatial setups of greenhouse and mesocosm studies (Garssen, Baattrup‐Pedersen, Voesenek, Verhoeven, & Soons, 2015; Garssen, Verhoeven, & Soons, 2014; Webb, Wallis, & Stewardson, 2012).

Plants need specific adaptations, like adventitious root formation or aerenchyma to survive prolonged flooding (Voesenek, Colmer, Pierik, Millenaar, & Peeters, 2006). Differences between species traits determine their flooding tolerance and create a clear zonation along riparian elevation gradients (Fraaije, Ter Braak, Verduyn, Verhoeven, & Soons, 2015; Van Eck, Lenssen, Van De Steeg, Blom, & De Kroon, 2006; Webb et al., 2012). Therefore, after changes in flooding regime, drought‐ or flood‐tolerant species will be replaced by others that are better adapted to the new conditions (Ström, Jansson, Nilsson, Johansson, & Xiong, 2011) and induce changes in the riparian zonation (Antheunisse & Verhoeven, 2008; Banach et al., 2009; Ström, Jansson, & Nilsson, 2012) and functioning (Lake, Bond, & Reich, 2007; Sarneel & Veen, 2017). However, while it is generally accepted that vegetation responses mostly are slow and that some intermediate stage may persist for a reasonable amount of time (Baastrup‐Spohr, Sand‐Jensen, Olesen, & Bruun, 2017; Hasselquist et al., 2015; Nilsson et al., 2015b), we lack understanding of how and how fast responses occur (Webb et al., 2012). Less is known about how riparian plants survive drought events, but root and leaf traits are thought to play a crucial role (Li et al., 2014). In a meta‐analysis, Garssen et al. (2014) conclude that a drought events longer than one month affect riparian seedling survival and plant growth negatively. Furthermore, extreme drought events have been shown to induce sudden changes in biodiversity by causing plant mortality, which is thought more likely in warmer than in colder climates (Garssen et al., 2014).

Some indirect empirical evidence indicates that the duration and species composition of transitional stages relate to the strength of environmental filtering of the new conditions (e.g. stress level) and to initial vegetation characteristics (historic factors). For instance, plant species distribution along the river Rhine was found to reflect an extreme flood 14 years in the past (Van Eck et al., 2006). This suggests (a) that the vegetation drastically and quickly changed after this strong disturbance, and (b) that recovery was slow during the low‐stress period afterwards. This aligns well with the hypothesis that the magnitude of stress affects both the magnitude of change and the rate of recovery or convergence to new conditions, but long‐term experimental studies are needed to shed light on how mortality and invasion could drive such changes. The rate of vegetation change can further be driven by other time and soil moisture‐dependent processes such as the speed of nutrient release due to changed redox potentials (Beltman, Willems, & Gusewell, 2007) or the rate of groundwater‐table drawdown in case of drought events (Froend & Sommer, 2010). In addition to abiotic conditions, vegetation composition can also determine the amount and rate of change. This was demonstrated within the Jena biodiversity experiment, where plant performance directly after flooding was less affected in high diversity plots (Wright et al., 2017).

Recent work, however, suggests that stochastic factors, like historic contingency, can interfere with the straightforward replacement of sensitive with tolerant species and can result in alternative transitional stages, or alternative stable states (Fukami & Nakajima, 2011; Maren, Kapfer, Aarrestad, Grytnes, & Vandvik, 2018; Sarneel, Kardol, & Nilsson, 2016; Stuble, Fick, & Young, 2017; Vannette & Fukami, 2014). For instance, in heathlands, replicate turfs followed different routes before converging to a similar vegetation type 7 years after fire disturbance (Maren et al., 2018). The rapidly developing theoretical framework behind alternative transitional stages suggests that the duration of the transient period could be correlated with mortality (Fukami & Nakajima, 2011). This is because mortality, induced by the changed stress level or by changed biotic interactions, will open patches which allow invasion of potentially better adapted species (Catford & Jansson, 2014), but field proof is lacking. To explore pathways of change after various changes in flooding regimes, we set up a long‐term experiment and transplanted vegetation turfs full‐factorial between three riparian elevations that correspond to different vegetation zones (upland border, middle and low elevation). Given the strong environmental forcing in riparian zones and the clear vegetation zonation within them, we hypothesize that the transplanted vegetation will eventually converge towards the new (target) vegetation, potentially via alternative transitional stages. Since target vegetation occurs directly adjacent to the transplanted turfs, dispersal will not strongly limit the rate of vegetation change. We hypothesize that due to the absence of strong dispersal barriers, the rate of vegetation change will be determined by factors that determine mortality (i.e. stress level) and or invasion (germination, establishment) and that response rates of individual species reflect their flooding tolerance, which we quantified using Ellenberg moisture values.

## 2 MATERIALS AND METHODS

### 2.1 Study area

**Figure 1:**
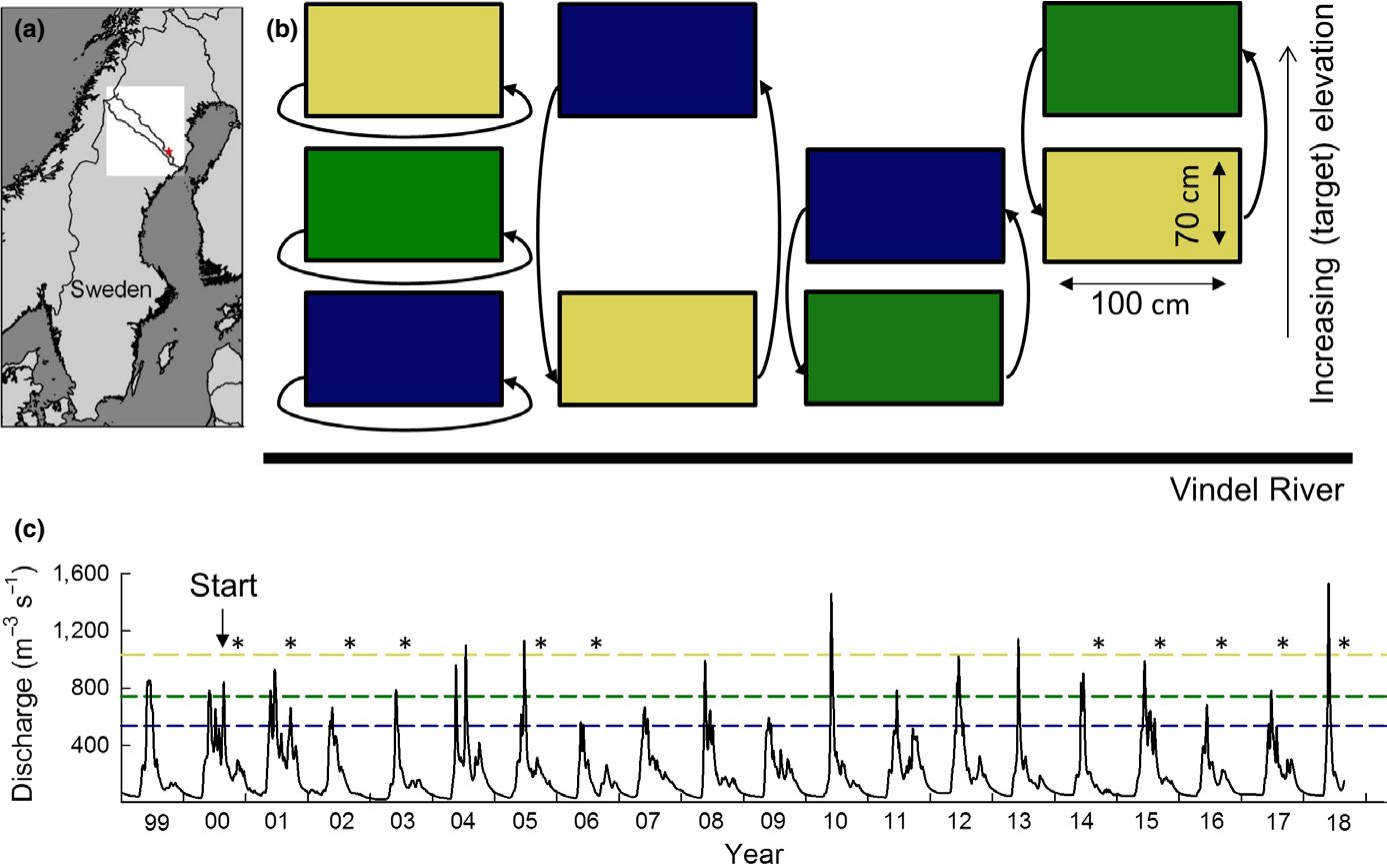
Experimental settings. (a) Location in the Ume‐Vindel River catchment. The red star indicating the experimental site (Strycksele). (b) Experimental design of turf transplantation (*n* = 8). The arrows indicate how turfs were transplanted, with colours indicating initial elevation (yellow upland border, green middle and blue low elevation). (c) Discharge 25 km downstream of Strycksele (at the Granåker gauge), with dashed lines indicating when different elevations are flooded (colours as in b). Asterisks indicate when vegetation surveys were performed [Colour figure can be viewed at http://wileyonlinelibrary.com]

The Vindel River is a ca. 455‐km‐long, free‐flowing river that flows from the Norwegian border across Sweden into the Baltic Sea (Figure 1a). The Vindel River has a mean annual discharge of 188 m^3^ s^−1^ at the point where it joins the Ume River (2000–2015, Swedish Meteorological and Hydrological Institute, SMHI), 30 km upstream from the mouth. Its flow is characterized by spring floods in May or June, declining water levels during the rest of the year, and some rain‐fed, smaller floods during late summer and autumn. Induced by the flooding and associated soil moisture gradient riparian vegetation along the river is strongly zoned, with dense graminoid belts at the lowest elevation, willow shrubs at an intermediate elevation, forbs and grasses towards the upland border and mixed birch and pine forest when moving towards terrestrial ecosystems (Ström et al., 2011). We selected a 250 m long and circa 35 m wide floodplain meadow along a tranquil reach near the village Strycksele (Figure 1a, 64^o^22′N, 19^o^22′E), which was used for haymaking until 1950–1960 but left unused since then. To characterize the soil conditions, soil samples were taken at high and low elevations in 2000 (*n *=* *3 at each elevation) and on 23 May 2014 (*n *=* *8) and analysed for pH by shaking 5 g fresh soil for a few hours in 50 ml demineralized water. Soil C:N concentration was measured by combustion. Soils collected in August 2014 were dried 48 h drying at 104°C to determine their moisture content as percentage weight loss.

### 2.2 Experimental setup

In June 2000, a total of 72 soil turfs were excavated, in eight replicated random blocks spread across the bank in Strycksele. Each block consisted of nine turfs (70 × 100 cm × 20 cm depth), three at the upper border, located on the floodplain grassland, close to the forest edge, three at middle elevation on grassland patches within the zone dominated by *Salix* shrubs and three at low elevations, within the dense graminoid zone (Figure 1b). The average elevation difference was 1.09 m ± 0.05 S.E. between turfs on the upland border and middle elevation and 0.77 m ± 0.03 S.E. (*n *=* *24) between middle and low elevations. Turf depth (ca. 20 cm) was deeper than the main rooting zone and included whole plants. At each elevation one turf was rotated 180° and carefully put back on the same location as a control for excavation (Figure 1b). The other turfs were placed in cradles and transplanted to their new target elevations. The (rotated) control turfs are referred to as “initial” and “target” vegetation relative to the transplanted ones. The turfs that were transplanted to higher elevation simulated the effect of decreased flooding regimes, while the turfs that were moved to lower elevations simulated the effects of increased flooding. In 2001 and 2002, litter was added to half of the blocks. However, we treated all turfs as replicates, since Ström et al. (2011) did not find any effects of litter addition after 2003.

### 2.3 Measurements

In each turf, we determined species composition once a year, always in August. Vegetation was sampled yearly from 2000 to 2003, 2005, 2006 and yearly from 2014 to 2018. In August 2014 and 2017, we surveyed only the extremes (upland border and lowest elevation) due to time constrains. We quantified vegetation cover using the pin‐point method, with 16 points in a 50 × 50 cm quadrat, starting 10 cm from the plot edge. For each species we recorded their cover as the number of pins that this species touched (thus with a maximum cover of 16). Species that were present in the quadrat but did not touch a pin were also recorded. We followed the taxonomy in Krok and Almquist (1994) and combined the following species to one taxon each to minimize observer effects: *Agrostis stolonifera + A. canina, Carex juncella* + *C. nigra*,* Hieracium* spp., *Hierochloë hirta* + *H. odorata*,* Luzula multiflora* + *L. sudetica*,* Poa* spp., *Salix* spp. and *Taraxacum* spp.

At each sampling occasion in August during the period 2000–2006 and in 2016, a biomass sample (15 × 20 cm) was taken in each turf, next to the vegetation survey. All samples were dried at least 48 h at 60°C and weighted.

### 2.4 Data analysis

Trends in vegetation development were analysed using nonmetric multidimensional scaling (NMDS) and the Vegan 2.4‐3 package (Oksanen et al., 2012) in R version 3.2.5 (R Core Team, 2016). We used the pin‐point scores of all years and all replicate turfs as input and species that were present in a turf but did not touch a pin were given the value 0.5. We used a Bray–Curtis distance and added “no.share = 0.1” to deal with the low overlap of species in some turfs. We extracted four dimensions and found convergence after 199 runs, with a stress of 0.105. We used two‐way ANOVA and Tukey's post hoc tests to test if the NMDS scores of the transplanted turfs at the first two axes differed from the controls at initial and target elevation.

To be able to compare the movement in NMDS space and the degree to which convergence to the target conditions had occurred in the different treatments on a similar scale, we standardized the moved distances by total trajectory length of a treatment. To calculate this, we first calculated both the shortest Euclidean distance between a turf (*i*) and the centroids of controls at the initial (Dist_INi_) and at target vegetation (Dist_TAi_). These distance measures were summed to obtain the total trajectory length between initial and target vegetation. Subsequently, we divided Dist_INi_ by the total trajectory length to obtain the relative distance (rDist) moved away from the initial vegetation composition. In summary, rDist = Dist_INi_/(Dist_TAi_ + Dist_INi_). This allows comparing the degree of converging to target conditions in trajectories that differed in absolute distance in NMDS space.

The rate at which turfs moved along the total trajectory between initial and target elevation was expressed as the difference in rDist at two sampling occasions, divided by the number of years between those sampling occasions. Thus, the rate of change = abs(rDist_t+1_ − rDist_t_)/(Year_t+1_ − Year_t_). Both rDist (%) and rate of change (% yr^−1^) were calculated per turf and averaged per treatment and sampling interval combination (*n *=* *8).

For each species in each turf, we calculated the rate of cover change as the regression coefficient of the pin‐point scores versus time (years). To deal with the infrequent and low occurrence of rare species, we selected species that (a) occurred in three or more years in a certain turf, and (b) increased to or decreased from a cover of three or more. We averaged values per species for the three treatments that were moved in the same direction along the elevation gradient and correlated those with the Ellenberg value for moisture, which we obtained from the Online British Flora (https://www.brc.ac.uk/plantatlas/). We could not obtain an Ellenberg value for only eight species, of which only one species was frequently occurring in the turfs (*Salix* spp). To not overlook this species, we gave this species the Ellenberg value of *Salix phylicifolia* (Ellenberg moisture value = 8) as this species was most frequently reported within this aggregated species group.

Colonization and mortality events drive vegetation change. However, there may be a mismatch between the two and sometimes mortality may be more important for changes in the vegetation, whereas other vegetation changes are driven by colonization. As a measure of such demographic changes within the vegetation, we calculated the summed relative cover change per species (Damgaard, Merlin, & Bonis, 2017). For each species in each turf, we calculated the difference in cover (pin‐point scores) between two consecutive years. For each turf, we divided the sum of all the negative values by the total sum of cover in that turf in the first year of the time interval. Such cover loss represents mortality and can provide open space for invasion, for example, of better adapted species (Damgaard et al., 2017). Likewise, the sum of the positive values was divided by the total cover sum to obtain a measure of rate of invasion or species increase. These proxies were averaged per treatment and time interval and correlated with the relative rate of change in NMDS space.

## 3 RESULTS

### 3.1 Environmental conditions

Flooding duration roughly halved with each step from low elevation towards the upland border. That is, in our study period, turfs at low elevation were flooded yearly for about 21.0 ± 2.5 S.E. days (Swedish Meteorological and Hydrological Institute, Figure 1c, *n* = 19), while at the middle elevation, turfs were flooded roughly for 2 out of 3 years, for 10.6 ± 1.3 S.E. days per flooding event (*n *=* *14). Upland turfs were flooded only five times and flood events lasted on average 5.2 ± 1.35 S.E. days (*n *=* *5). Floods occurred predominantly in spring, but in 2000, 2001 and 2015, the lowest turfs were also flooded during the summer. Soil moisture content in August 2014 decreased from 36.34% ± 1.54 S.E. at low elevation to 24.47% ± 1.15 S.E. at the upland border.

The average pH (5.59 ± 0.06 S.E., *n *=* *16, 2014) remained unchanged over the study period, but the average nitrogen (N) and carbon (C) concentrations increased (N: from 0.18 ± 0.01 S.E. gg^−1^ dry soil in 2000 *n *=* *6, to 0.30 ± 0.03 S.E. gg^−1^ dry soil in 2014 *n *=* *16; C: from 2.72 ± 0.19 S.E. gg^−1^ dry soil to 4.44 ± 0.38 S.E. gg^−1^ dry soil). In 2014, neither pH, soil nitrogen nor soil carbon concentrations differed between elevations (for details see Sarneel & Veen, 2017).

### 3.2 Biotic responses

The lowest riparian elevation was inhabited by a productive (485 ± 52 S.E. gm^−2^; *n *=* *8, Figure S1) and species‐poor vegetation (3.4 ± 0.2 S.E. species per plot; *n *=* *8, See Figure S1), while the upland border was less productive (287 ± 33 S.E. gm^−2^, *n *=* *8) and more species rich (9.6 ± 0.82 S.E., *n *=* *8).

**Figure 2:**
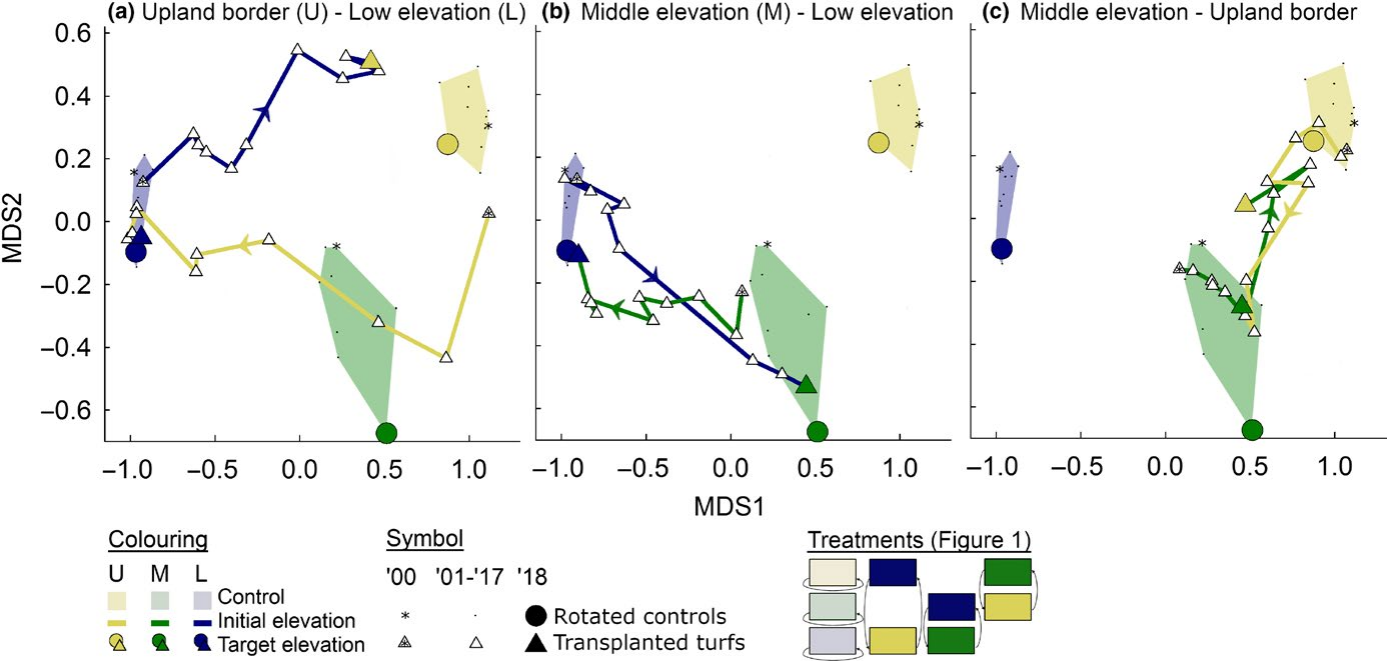
Vegetation changes over time in the different transplantation experiments. The movement over time of turfs that were moved between (a) upland and low, (b) middle and low and (c) middle and upland elevations in nonmetric multidimensional scaling (NMDS) space. Each dot is the centroid of eight replicate turfs

**Figure 3:**
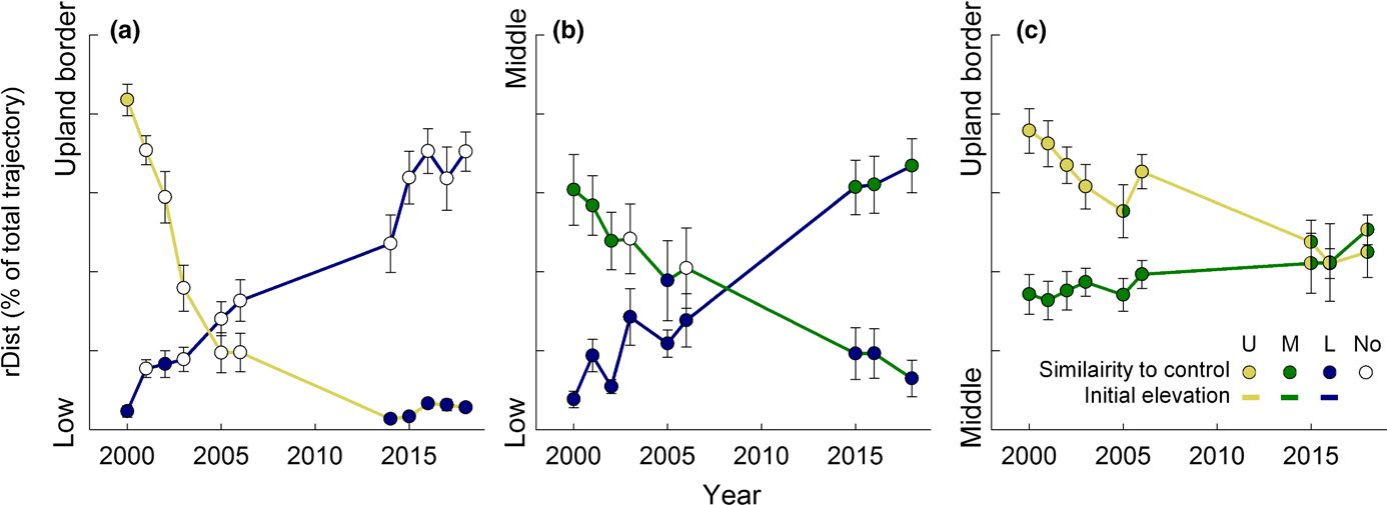
The degree of convergence between initial and target vegetation as the relative distance to control vegetation for the pair that moved between (a) upland border and low elevation, (b) middle and low elevation and (c) upland border and low elevation. Note that the y‐axis of the different panels differ. Values on the *y*‐axis are the relative distance of the treatment turfs to the control centroids indicated on the axis (20% increments per thick mark). Each dot is the mean of eight replicates, and the filling indicates if the NMDS scores of the turfs were significantly (in)different to one or both control turfs

**Figure 4:**
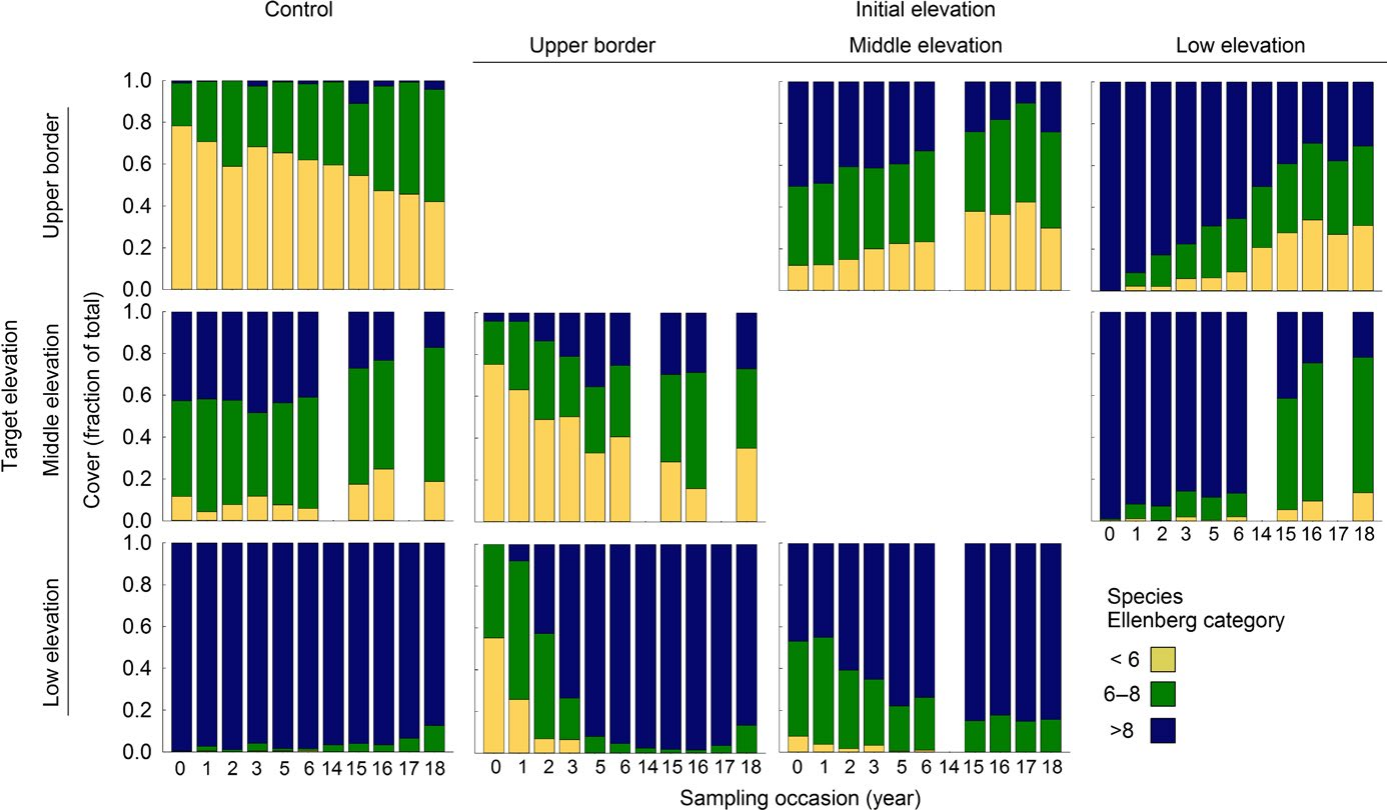
Proportion of the plant community with specific moisture preferences (Ellenberg moisture values) in the control (left column) and transplanted turfs based on the pin‐point scores. Panels are grouped per initial elevation (columns) and target vegetation (rows). Each bar is the average of eight replicates

The NMDS diagram shows clearly that directly after transplantation, the vegetation composition of the transplanted turfs resembled the vegetation at the initial elevation (Figure 2 and Table S1 for statistics). Over time, major species shifts occurred in the transplanted turfs, while the control turfs did not change much (Figure 2). Eventually in 2018, the species composition of most turfs resembled the vegetation of the target elevation. As an important exception, turfs that were moved from low elevation to the upland border remained significantly different from their target vegetation (Figure 2, NMDS1 scores; Tukey's post hoc; *P *<* *0.001 *df *= 63; Table S1). For these transplanted turfs, their movement in NMDS space during the last 4–5 years (Figures 2a, 3a), may even suggest the development of an alternative stable state, as it does not seem to move closer to the target centroid. Here, low‐elevation species (mainly *Carex acuta* and *Calamagrostis canescens*) were still present with relative high cover (Figure 4).

### 3.3 Trajectories of vegetation development

Most trajectories through NMDS space followed a rather direct route between the centroids of the initial and target control turfs, but examples of alternative trajectories were found. For instance, the trajectory of the turfs that were moved from the upland border to low elevations, transiently resembled mid‐elevation vegetation (Figure 2a). The trajectory of turfs that were moved in the opposite direction (from low elevation to the upland border) was completely different (Figure 2a). The turfs that were moved from and towards the middle elevation followed a more similar route in NMDS space when compared to their counterparts that were moved in the opposite direction (Figure 2b, c).

### 3.4 Rate of change

Most turfs appear to change rather linearly, that is, with a constant rate over time, except for the turfs that were moved from high to low elevations where changes were faster during the first years of the study (Figure 3), and slower during later years. The turfs that were moved down in elevation generally changed faster and came to resemble the target vegetation earlier compared to the turfs that were moved in the opposite direction (Figure 3). The vegetation in the turfs that were moved from middle to low elevations were the first to convert from initial the vegetation to the target elevation (after six growing seasons). In some cases, we observed retrogression, where turfs became more similar to the initial vegetation (Figure 3b,c), but these changes did not last longer than 1 year.

Changes in the proportions of species with affinity for a certain soil moisture level confirmed that turfs transplanted from the upland border to low elevation adjusted more quickly to the target vegetation than turfs transplanted from low elevation to the upland border (Figure 4). In 2018, species with a high affinity for soil moisture (Ellenberg values > 8) contributed on average 30.7% ± 10.1 S.E. of the total cover of the turfs that moved from low elevation to the upland border, whereas such species are almost absent in the control turfs at the upper border (Figure 4). Cleary, the relative cover of species with intermediate affinity for soil moisture increased in turfs that moved from the upper border to low elevation, but after ca. three to four growing seasons their proportion decreased to values comparable to the control turfs at the target elevation (Figure 4).

### 3.5 Species driving changes in vegetation patterns

**Figure 5:**
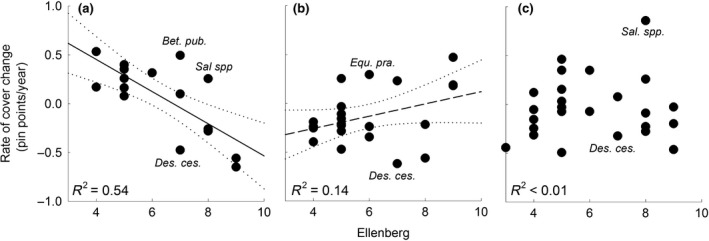
Rate of cover change per species in treatments where turfs were moved towards (a) higher and (b) lower elevations and (c) remained at their own elevation in relation to their Ellenberg value for moisture. Each point is the mean of one species in three treatments. Indicators of succession are indicated with abbreviated species names (*Betula pubescens*,* Deschampsia cespitosa*,* Equisetum pratense* or *Salix* spp, which were assigned the Ellenberg value of the common *Salix phylicifolia*). Lines indicate regression lines and their 95% confidence interval

Across all the transplanted turfs we observed that species that were best adapted to the new (target) conditions were the ones that appeared and increased vigorously and fast in cover, while the least adapted species decreased. That is, in turfs that were moved to less flooded conditions, the cover of species with high Ellenberg values decreased quickly while species with low Ellenberg values increased (*F*_1,15_ = 17.79, *P *=* *0.001, *R* ^2^ = 0.54, Figure 5a). In contrast, in turfs that were moved to more frequently flooded conditions, species with low Ellenberg values disappeared and the cover of species with high Ellenberg values increased, but this correlation was not significant (*F*_1,20_ = 3.16, *P *=* *0.09, *R* ^2^ = 0.14, Figure 5b). We observed that species that are associated with succession changed disproportionally to their Ellenberg value, for example, *Betula pubescens*,* Salix* spp. and *Equisetum pratense* increased, while *Deschampsia cespitosa* decreased. These trends were also observed in the control turfs where the Ellenberg value and the species‐specific rate of cover change did not correlate (*F*_1,22_ = 0.12, *P *=* *0.735, *R* ^2^ < 0.01, Figure 5c).

**Figure 6:**
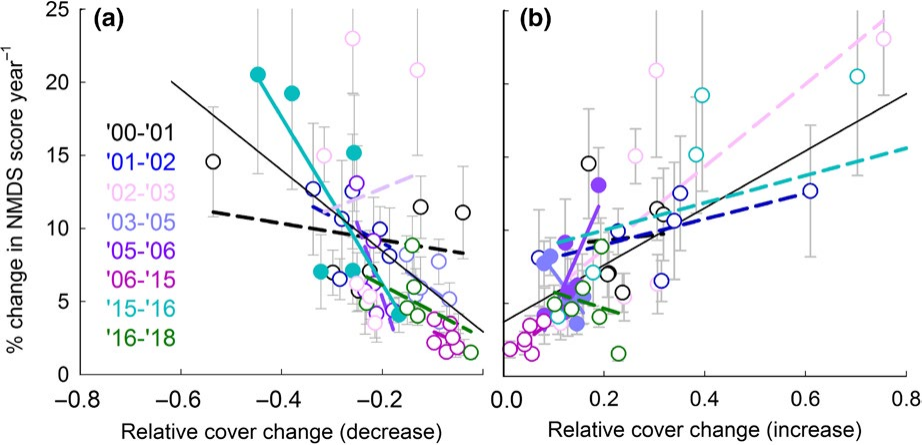
Correlation between (a) the relative decrease in cover and (b) the relative cover increase and the rate of change in NMDS coordinates. One point is the average of eight replicates, and lines are fitted across the six transplanted treatments within one year. Solid lines and associated filled symbols indicate significant correlations, with the black thin line showing the overall trend. Grey bars indicate S.E

At the community level, the cover loss per year of all the species that decreased in cover in a turf correlated with the rate of change in NMDS space (Pearson ρ = −0.59 *t*_46_ = −4.92, *P *<* *0.001; Figure 6). The relative cover decrease exceeded the cover increase in the turfs that were moved to lower elevations during the first years, which could lead to the creation of open space (Figure S2). The relative cover increase also correlated with the change in NMDS space (Pearson ρ = 0.67, *t*_46_ = 6.07, *P *<* *0.001), showing that (other) species increased in cover and invaded the turf. These correlations were rather consistent among years, although only significant in some years (Figure 6; relative decrease 2015–2016, Pearson ρ = −0.81, *t*_4_ = −2,76, *P *=* *0.051; relative increase 2003–2005, Pearson ρ = −0.85, *t*_4_ = 3.17, *P *=* *0.034; and 2005–2006 Pearson ρ = 0.81, *t*_4_ = 2.80, *P *=* *0.049).

## 4 DISCUSSION

We found that past flooding regimes can still affect community composition after a period of 19 growing seasons. Although individual species increased and decreased predictably following their preferences and adaptations for soil moisture, strikingly different vegetation communities were observed for intermediate transition stages. These results provide rare experimental proof that alternative transient trajectories and stable states can occur under field conditions, which is an important step towards understanding the drivers behind community response to global change.

In many zoned ecosystems, such as riparian and tidal ecosystems, the lower boundary of a species is hypothesized to be determined by stress tolerance, while the upper border is formed by competition (Bertness & Callaway, 1994). We therefore assume that for turfs that were moved to more frequently flooded locations, vegetation changes could be driven by (sudden) plant death opening space for invasion, while when moving to the higher, drier locations, where mortality is lower, competition could be the driving force (Maren et al., 2018). Our observations support this idea, with vegetation responses to moving downward in elevation proceeding more rapidly compared to moving to higher elevations, and the correlation between rate of vegetation change and our proxies for mortality being steeper than with our proxies for invasion. Increased flooding stress levels likely had more direct and larger effects on vegetation composition than increased competitive pressure, thus leading to more gradual changes at higher elevations. The observation that the fastest convergence to target vegetation (six growing seasons), the turfs that moved from the middle to the low elevation, may be due to a combination of increased stress levels at the target elevation and the prior presence of low‐elevation species at the middle elevation.

Since mortality appeared to be a driver of fast changes, it was somewhat surprising that the relationship between Ellenberg moisture values and species‐specific cover change was not significant in the turfs that were moved to elevations with higher flooding stress levels. It may be that, rather than a gradual change, flooding stress may only allow species with certain moisture tolerance thresholds to persist (Garssen et al., 2015), which would result in nonlinear relations. The correlation between Ellenberg value and changes in species cover in the turfs that were moved to dryer locations may thus be interpreted as the effect of moisture on competitive strength of species rather than on their survival. Although stress‐driven mortality may result in sudden changes, competition may allow species with an intermediate competitive strength to persist at lower densities, making the abundance change with an intermediate rate.

In sum, we observed that the rate of change towards target vegetation was both driven by factors leading to species disappearance (e.g. mortality; Fukami & Nakajima, 2011) and to factors that enhance species growth or invasion (dispersal, establishment from the seed bank or clonal expansion) such as suggested by the enhanced germination in turfs transplanted to high elevations (Sarneel, Bejarano, Van Oosterhout, & Nilsson, 2019). Our study system likely represents rather fast invasion compared to other systems, since dispersal limitations for our transplanted turfs are low, and it is speculated that riparian systems are relatively easy to invade compared to other systems (Catford & Jansson, 2014). Hence, the slow response observed in our study could still be at the fast end of the continuum compared to other ecosystems with similar climatic conditions (Hasselquist et al., 2015). Such an interaction between climate and ecosystem properties on the slow rates of change is important to consider when evaluating environmental impact assessments or restoration efforts in other systems or climatic conditions (Garssen et al., 2014).

We not only observed differences in rate of change, we also observed alternative, often transient, intermediate stages. That is, in the treatment that experienced the strongest increase in stress and disturbance level (the turfs that moved from the upper border to low elevation) we saw a temporal increase in mid‐elevation species. We hypothesize that the mid‐elevation species could have had a temporal advantage (e.g. of increased soil moisture) before low‐elevation species outcompeted them. Additionally, the relatively mild spring flood in 2002 may have stimulated mid‐elevation species at low elevation. Such factors could also have steered the development of alternative trajectories in the turfs that were moved between upland and low elevations, showing that specific historic events (e.g. the low spring flood in 2002) may disproportionally affect vegetation development (Stuble et al., 2017). Stochastic events may also have been responsible for the retrogression, as relatively dry or wet years may have stimulated certain species. For instance, the retrogression in 2006 for turfs transplanted from the upland border to middle elevation, correspond with the low spring flood that year.

Only very few studies have investigated the effect of delayed or gradual vegetation response for the functioning and ecosystem services in riparian zones (Lake et al., 2007). For riparian soil functions, decomposition was shown to be intermediate for soils that had recently experienced a change in flooding regime (Sarneel & Veen, 2017), which is in line with the observed sensitivity of microbial communities to soil moisture legacies (Hawkes & Keitt, 2015). The effects of other vegetation‐mediated changes in riparian functioning such as bank stabilization, water purification or litter subsidies to the aquatic food web remain to be investigated (Lake et al., 2007; Polvi & Sarneel, 2018).

When predicting the effect of environmental and climatic changes on the rate and trajectory by which convergence to the new conditions takes place, our study suggests that rates of initial mortality, and later invasion rates, are import drivers. Those, in their turn, depend on the initial vegetation composition and the difference between the old and new environmental conditions. (Lake et al., 2007; Nilsson et al., 2015a). An additional implication for restoration studies is that there may be multiple pathways of ecosystem recovery, which may differ from the trajectory of degradation. Such hysteresis effects (Fukami & Nakajima, 2011; Lake et al., 2007; Stuble et al., 2017) may be caused by differences in the rates of decline and extinction in relation to probability of colonization and establishment, as reported in this study. Finally, studies projecting vegetation responses to climate change face the triple challenges of predicting the time needed for vegetation adjustment, the likely pathways of change and the fact that controls may represent moving targets (Arft et al., 1999; Olsen, Topper, Skarpaas, Vandvik, & Klanderud, 2016). These uncertainties can be minimized by knowledge of mortality risks in response to the abiotic conditions under change, competitive interactions among species in the community in focus and the probability of new species colonizing communities (Catford & Jansson, 2014; Fukami & Nakajima, 2011; Olsen et al., 2016).
