# Supplementary material for: Alternative transient states and slow plant community responses after changed flooding regimes
Source: Glob Chang Biol. 2019 Jan 30;25(4):1358–67. doi: 10.1111/gcb.14569 (PMC6849759; doi:10.1111/gcb.14569)
Supplement: Supplementary file 1 [file GCB-25-1358-s001.docx]

**Table S1:** Post hoc test on the difference in NMDS score for the first two axis. Comparisons were done between pairs of turfs that were moved between two elevations and the control turfs at those elevation. Shaded cells indicate the comparison to control turf at the target elevation and not shaded cells to the initial elevation. Bold *P-*values indicate significant differences.

(**a**) Turfs that were moved between the upland border and low elevation

|  | | From low elevation to upland border | | | | From upland border to low elevation | | | |
| --- | --- | --- | --- | --- | --- | --- | --- | --- | --- |
| Control turf |  | NMDS1 | | NMDS2 | | NMDS1 | | NMDS2 | |
|  | Year | Difference | P | Difference | P | Difference | P | Difference | P |
| Upland border | 2000 | **-2.043** | **<0.001** | -0.179 | 0.702 | -0.002 | 1.000 | -0.278 | 0.352 |
|  | 2001 | **-1.741** | **<0.001** | -0.074 | 0.962 | **-0.250** | **0.001** | **-0.787** | **<0.001** |
|  | 2002 | **-1.701** | **<0.001** | -0.090 | 0.950 | **-0.638** | **<0.001** | **-0.655** | **0.003** |
|  | 2003 | **-1.604** | **<0.001** | -0.273 | 0.340 | **-1.233** | **<0.001** | **-0.552** | **0.009** |
|  | 2005 | **-1.478** | **<0.001** | -0.067 | 0.955 | **-1.682** | **<0.001** | -0.342 | 0.065 |
|  | 2006 | **-1.383** | **<0.001** | 0.089 | 0.901 | **-1.680** | **<0.001** | -0.314 | 0.098 |
|  | 2015 | **-0.574** | **0.002** | 0.013 | 1.000 | **-1.791** | **<0.001** | -0.417 | 0.113 |
|  | 2016 | **-0.526** | **0.001** | 0.116 | 0.881 | **-2.009** | **<0.001** | -0.420 | 0.056 |
|  | 2018 | **-0.454** | **0.001** | 0.262 | 0.330 | **-1.808** | **<0.001** | -0.296 | 0.231 |
| Low elevation | 2000 | -0.052 | 0.543 | 0.033 | 0.997 | **-2.093** | **<0.001** | 0.132 | 0.856 |
|  | 2001 | **-0.289** | **<0.001** | -0.067 | 0.971 | **-1.781** | **<0.001** | **0.646** | **0.001** |
|  | 2002 | -0.307 | 0.140 | -0.110 | 0.914 | **-1.371** | **<0.001** | **0.455** | **0.053** |
|  | 2003 | **-0.316** | **0.047** | -0.055 | 0.986 | **-0.686** | **<0.001** | 0.224 | 0.507 |
|  | 2005 | **-0.540** | **<0.001** | -0.037 | 0.992 | **-0.336** | **0.014** | 0.238 | 0.290 |
|  | 2006 | **-0.660** | **<0.001** | -0.189 | 0.477 | **-0.363** | **0.018** | 0.214 | 0.372 |
|  | 2015 | **-1.207** | **<0.001** | -0.379 | 0.170 | 0.011 | 1.000 | 0.051 | 0.992 |
|  | 2016 | **-1.466** | **<0.001** | **-0.591** | **0.004** | 0.017 | 0.999 | -0.056 | 0.984 |
|  | 2018 | **-1.385** | **<0.001** | **-0.605** | **0.003** | -0.031 | 0.992 | -0.046 | 0.991 |

(**b**) Turfs that were moved between middle and low elevation

|  | | From low elevation to middle elevation | | | | From middle to low elevation | | | |
| --- | --- | --- | --- | --- | --- | --- | --- | --- | --- |
| Control turf |  | NMDS1 | | NMDS2 | | NMDS1 | | NMDS2 | |
|  | Year | Difference | P | Difference | P | Difference | P | Difference | P |
| Middle elevation | 2000 | **1.199** | **0.001** | -0.209 | 0.215 | -0.150 | 0.941 | -0.151 | 0.489 |
|  | 2001 | **0.979** | **0.001** | -0.173 | 0.640 | -0.120 | 0.952 | -0.283 | 0.236 |
|  | 2002 | **1.095** | **0.001** | -0.303 | 0.190 | -0.374 | 0.434 | -0.069 | 0.965 |
|  | 2003 | **0.748** | **0.002** | -0.244 | 0.251 | **-0.495** | **0.048** | -0.071 | 0.945 |
|  | 2005 | **0.950** | **0.000** | **-0.385** | **0.040** | **-0.761** | **0.001** | 0.106 | 0.862 |
|  | 2006 | **0.885** | **0.000** | -0.341 | 0.099 | **-0.685** | **0.004** | 0.112 | 0.859 |
|  | 2015 | 0.330 | 0.172 | 0.147 | 0.927 | **-1.305** | **0.000** | 0.050 | 0.997 |
|  | 2016 | 0.265 | 0.505 | 0.214 | 0.832 | **-1.362** | **0.000** | -0.022 | 1.000 |
|  | 2018 | 0.067 | 0.982 | -0.145 | 0.880 | **-1.412** | **0.000** | **0.561** | **0.037** |
| Low elevation | 2000 | 0.004 | 1.000 | 0.026 | 0.995 | **1.045** | **0.003** | **-0.386** | **0.005** |
|  | 2001 | -0.090 | 0.979 | 0.120 | 0.844 | **0.949** | **0.001** | **-0.577** | **0.003** |
|  | 2002 | 0.002 | 1.000 | 0.005 | 1.000 | **0.719** | **0.031** | -0.377 | 0.071 |
|  | 2003 | -0.238 | 0.557 | 0.115 | 0.808 | **0.490** | **0.051** | **-0.431** | **0.012** |
|  | 2005 | -0.216 | 0.633 | 0.099 | 0.884 | 0.405 | 0.136 | **-0.378** | **0.045** |
|  | 2006 | -0.318 | 0.310 | 0.145 | 0.739 | **0.518** | **0.036** | -0.374 | 0.061 |
|  | 2015 | **-1.085** | **0.000** | 0.523 | 0.150 | 0.110 | 0.894 | -0.327 | 0.528 |
|  | 2016 | **-1.304** | **0.000** | 0.378 | 0.455 | 0.207 | 0.692 | -0.186 | 0.882 |
|  | 2018 | **-1.412** | **0.000** | 0.433 | 0.166 | 0.066 | 0.984 | -0.017 | 1.000 |

c) Turfs that were moved between middle elevation and upland border

|  | | From middle elevation to upland border | | | | From upland border to middle elevation | | | |
| --- | --- | --- | --- | --- | --- | --- | --- | --- | --- |
| Control turf |  | NMDS1 | | NMDS2 | | NMDS1 | | NMDS2 | |
|  | Year | Difference | P | Difference | P | Difference | P | Difference | P |
| Upland border | 2000 | **-1.034** | **0.006** | -0.465 | 0.074 | -0.048 | 0.998 | -0.087 | 0.963 |
|  | 2001 | **-0.951** | **0.001** | -0.519 | 0.169 | -0.077 | 0.986 | -0.156 | 0.919 |
|  | 2002 | **-0.829** | **0.012** | -0.532 | 0.171 | -0.197 | 0.857 | -0.029 | 0.999 |
|  | 2003 | **-0.773** | **0.006** | **-0.707** | **0.029** | -0.282 | 0.561 | -0.238 | 0.749 |
|  | 2005 | **-0.720** | **0.012** | -0.471 | 0.127 | -0.472 | 0.151 | -0.120 | 0.937 |
|  | 2006 | **-0.597** | **0.028** | -0.465 | 0.096 | -0.223 | 0.682 | -0.042 | 0.996 |
|  | 2015 | -0.220 | 0.728 | -0.473 | 0.403 | -0.348 | 0.368 | -0.639 | 0.165 |
|  | 2016 | -0.352 | 0.260 | -0.284 | 0.763 | -0.466 | 0.084 | -0.726 | 0.082 |
|  | 2018 | -0.402 | 0.202 | -0.204 | 0.883 | -0.423 | 0.167 | -0.526 | 0.255 |
| Middle elevation | 2000 | 0.134 | 0.965 | 0.084 | 0.968 | **-0.852** | **0.028** | -0.294 | 0.387 |
|  | 2001 | -0.011 | 1.000 | 0.085 | 0.985 | **-0.884** | **0.003** | -0.278 | 0.668 |
|  | 2002 | -0.087 | 0.985 | 0.024 | 1.000 | **-0.719** | **0.034** | -0.479 | 0.247 |
|  | 2003 | -0.162 | 0.874 | 0.020 | 1.000 | **-0.652** | **0.025** | -0.449 | 0.256 |
|  | 2005 | -0.132 | 0.927 | -0.118 | 0.940 | -0.380 | 0.312 | -0.469 | 0.129 |
|  | 2006 | -0.243 | 0.619 | -0.121 | 0.921 | **-0.617** | **0.022** | **-0.544** | **0.040** |
|  | 2015 | -0.146 | 0.900 | -0.269 | 0.804 | -0.017 | 1.000 | -0.103 | 0.986 |
|  | 2016 | -0.071 | 0.981 | -0.356 | 0.616 | 0.044 | 0.995 | 0.086 | 0.991 |
|  | 2018 | 0.042 | 0.997 | -0.716 | 0.070 | 0.062 | 0.989 | -0.394 | 0.500 |

***Figure S1****: Biomass (a-c) and species richness (d-f) over time in the turfs transplanted between elevations. Error bars indicate S.E., n = 8 for each point.*

*For description of the development of biomass and species richness under the period 2000 to 2006 see Ström, et al. ^14^. Biomass in 2016 was determined by target elevation (F_2,63_ = 33.81, P < 0.001), and not by origin (F_2,63_ = 0.15, P = 0.862) or an interaction between target elevation and origin (F_4,63_ = 0.23 P < 0.919). Species richness in 2016 was determined by target elevation (F_2,63_ = 60.05 P < 0.001), and not by origin (F_2,63_ = 0.23, P = 0.797) or an interaction between target elevation and origin (F_4,63_ = 0.78 P < 0.542).*

***Figure S2:*** *Net vegetation change over time in turfs that were moved between a) upland border and low elevation, b) middle and low elevation and between c) upland border and middle elevation. Positive values indicate that species change is due to invasion whereas negative values indicate that species cover are on average decreasing. Colour indicate turf origin, with yellow for upland border, green for middle and blue for low elevation.*
